# Association between Insomnia Symptoms and Hemoglobin A_1c_ Level in Japanese Men

**DOI:** 10.1371/journal.pone.0021420

**Published:** 2011-07-01

**Authors:** Yuko Kachi, Mutsuhiro Nakao, Takeaki Takeuchi, Eiji Yano

**Affiliations:** Department of Hygiene and Public Health, School of Medicine, Teikyo University, Tokyo, Japan; Genentech Inc., United States of America

## Abstract

**Background:**

The evidence for an association between insomnia symptoms and blood hemoglobin A_1c_ (HbA_1c_) level has been limited and inconclusive. The aim of this study was to assess whether each symptom of initial, middle, and terminal insomnia influences HbA_1c_ level in Japanese men.

**Methods:**

This cross-sectional study examined 1,022 male workers aged 22–69 years with no history of diabetes at a Japanese company's annual health check-up in April 2010. High HbA_1c_ was defined as a blood level of HbA_1c_ ≥6.0%. Three types of insomnia symptoms (i.e., difficulty in initiating sleep, difficulty in maintaining sleep, and early morning awakening) from the previous month were assessed by 3 responses (i.e., lasting more than 2 weeks, sometimes, and seldom or never [reference group]).

**Results:**

The overall prevalence of high HbA_1c_ was 5.2%. High HbA_1c_ was positively and linearly associated with both difficulty in maintaining sleep (P for trend  = .002) and early morning awakening (P for trend  = .007). More specifically, after adjusting for potential confounding factors, high HbA_1c_ was significantly associated with difficulty in maintaining sleep lasting more than 2 weeks (adjusted odds ratio, 6.79 [95% confidence interval, 1.86–24.85]) or sometimes (2.33 [1.19–4.55]). High HbA_1c_ was also significantly associated with early morning awakening lasting more than 2 weeks (3.96 [1.24–12.59]).

**Conclusion:**

Insomnia symptoms, particularly difficulty in maintaining sleep and early morning awakening, were found to have a close association with high HbA_1c_ in a dose-response relationship.

## Introduction

The prevalence of diabetes has been increasing in Japan, and the total number of Japanese with hyperglycemia was estimated to have risen from 14 million in 1997 to 22 million in 2007 [1]. Diabetes imposes a substantial burden in terms of premature mortality and health care costs [2]; therefore, the prevention of diabetes is recognized as an urgent public health priority.

A growing body of evidence suggests that insomnia is bidirectionally associated with diabetes. Insomnia can be secondary to diabetes because of diabetic complications or the psychological stress associated with diabetes management [3]–[5]. However, insomnia can also play an important role in the pathogenesis of diabetes. A recent systematic review and meta-analysis of 5 longitudinal studies showed that the major symptom of insomnia (i.e., difficulty in initiating or maintaining sleep) significantly predicts the risk for developing diabetes [6]. To help in the prevention and treatment of diabetes, it may be useful to clarify the effect of insomnia on glycemic status.

The blood level of hemoglobin A_1c_ (HbA_1c_) is perceived as the gold standard for monitoring glycemic control and is recommended as a diagnostic criterion for diabetes [7], [8]. However, only 2 cross-sectional studies have assessed the effect of insomnia symptoms on HbA_1c_ level. Knutson et al. [9] found a significant association between poor sleep quality and increased HbA_1c_ level in African-American patients with type 2 diabetes. In contrast, Nakajima et al. [10] did not find a significant association between insomnia symptoms and HbA_1c_ level in a Japanese non-patient population. Thus, the association between insomnia symptoms and HbA_1c_ level has been inconclusive.

The aim of the present study was to assess the association between insomnia symptoms and HbA_1c_ level in Japanese men. Although most previous studies have been limited by an inadequate assessment of insomnia symptoms [6] (e.g., they did not specify the time frame), we specified the duration of insomnia symptoms according to the criteria of the Diagnostic and Statistical Manual of Mental Disorders, Fourth Edition, Text Revision (DSM-IV-TR) [11].

## Materials and Methods

### Procedures and participants

This cross-sectional study was conducted in April 2010 as part of a Japanese company's annual health check-up, regulated by the Industrial Safety and Health Act. The participants in the study were full-time office workers at a general trading company in Tokyo. A self-administered questionnaire, along with a letter explaining the study's purpose and procedures, was distributed to all employees (n = 1,313) before the health check-up; 1,284 provided written informed consent to participate in the study and returned the questionnaire during the health check-up (response rate, 98%). Of these, 174 women were excluded because of the small sample size, and 88 men were excluded because of previously diagnosed or treated diabetes (n = 54) or missing data on any of the included variables (n = 34). A total of 1,022 men without previously diagnosed or treated diabetes were included in the analysis.

The present study was conducted according to the principles expressed in the Declaration of Helsinki. The protocol was approved by the Ethical Review Committee at Teikyo University School of Medicine.

### Outcome: high HbA_1c_ level

After an overnight fast, blood samples for HbA_1c_ were collected between 0800 and 1130 h. As the HbA1c level is relatively stable over time within individuals [7], differences in fasting duration may not influence the HbA_1c_ values. The value for HbA_1c_ (%) is estimated as an National Glycohemoglobin Standardization Program (NGSP) equivalent value (%) calculated by the formula HbA_1c_ (%) = HbA_1c_ (Japan Diabetes Society [JDS]) (%) +0.4%, taking into consideration the relational expression of HbA_1c_ (JDS) (%) measured by the previous Japanese standard substance and measurement methods and HbA_1c_ (NGSP).

High HbA_1c_ was defined as a blood level of HbA_1c_ ≥6.0%, which is the cutoff point for highly probable diabetes according to the definition of the Japan Diabetes Society [8]. This definition is in agreement with the International Expert Committee's recommendation that diabetes is to be diagnosed if the HbA_1c_ level is ≥6.5%, and that individuals with an HbA_1c_ ≥6.0% but <6.5% are likely to be at the highest risk for developing diabetes [7].

### Exposure: insomnia symptoms

Three types of insomnia symptoms from the previous month were assessed using the following questions: “Do you have difficulty falling asleep at night?” (difficulty in initiating sleep), “Do you wake up during the night after you have gone to sleep?” (difficulty in maintaining sleep), and “Do you wake up too early in the morning and have difficulty getting back to sleep?” (early morning awakening). For each question, 3 response options were offered: “lasting more than 2 weeks,” “sometimes,” and “seldom or never.” Insomnia symptoms lasting more than 2 weeks are in line with the DSM-IV-TR definition of insomnia [11]. These questions were adopted from our previous studies [12], [13], and the answers were confirmed by 2 male physicians specializing in psychiatry according to the DSM-IV-TR definition of insomnia [11].

### Covariates

Covariates were selected from established diabetes risk factors [14] and included age (continuous), body mass index (BMI; continuous), sleep duration (<6 or ≥6 hours per night), smoking status (never, past, or current), drinking habit (never, occasional, or daily), regular physical activity (yes or no), and family history of diabetes (yes or no). BMI was calculated by dividing the measured weight in kilograms by the square of the measured height in meters. Sleep duration of less than 6 hours has been shown to be associated with an increased risk for type 2 diabetes [6]. Regular physical activity was defined as continuing for at least 30 minutes, 2 times/week, for more than 1 year.

### Statistical analyses

First, the characteristics of the participants were compared across the 3 categories of each insomnia symptom. Second, the Mantel-Haenszel chi-square test for linear trend was used to determine the trend in the prevalence rates of high HbA_1c_ across the 3 categories of each insomnia symptom. Third, logistic regression models were used to assess the effect of each insomnia symptom on the risk for high HbA_1c_ after adjusting the covariates. Results were shown as odds ratios (ORs) with 95% confidence intervals (CIs). Three models were used, each with a different independent variable: model 1, difficulty in initiating sleep; model 2, difficulty in maintaining sleep; and model 3, early morning awakening. Finally, a sensitivity analysis was performed to evaluate the robustness of the results by treating blood levels of HbA_1c_ as a continuous variable. Multiple regression analyses were performed to assess the effect of each insomnia symptom on continuous HbA_1c_ levels after adjusting the covariates. There were no multicollinearity problems, and the residuals followed a normal distribution. All statistical tests were 2-sided, with a 5% significance level. All analyses were conducted using SAS Version 9.2 for Windows (SAS, Inc., Cary, NC).

## Results

Among the 1,022 participants included in the analysis, the mean and standard deviation values were 43.9±10.1 years (range, 22–69 years) for age, 24.1±3.3 kg/m^2^ for BMI, and 5.0±.4% for HbA_1c_ level. The distributions of insomnia symptoms were as follows: 1.5% of participants reported difficulty in initiating sleep lasting more than 2 weeks, 21.2% reported sometimes, and 77.3% reported seldom or never; 2.4% of participants reported difficulty in maintaining sleep lasting more than 2 weeks, 21.4% reported sometimes, and 76.2% reported seldom or never; and 3.4% of participants reported early morning awakening lasting more than 2 weeks, 29.6% reported sometimes, and 67.0% reported seldom or never. These distributions were almost identical to those from a previous Japanese nationwide study that used the same definitions for insomnia symptoms as those in our study [12]. Participants with a higher frequency of any type of insomnia symptoms were more likely to be older, to sleep less, and to not engage in regular physical activity (Table 1). In addition, participants with any type of insomnia symptoms lasting more than 2 weeks were more likely to be past smokers (Table 1).

**Table 1:** Characteristics of participants, by 3 categories of each insomnia symptom (n = 1,022).

	Difficulty in initiating sleep						Difficulty in maintaining sleep						Early morning awakening					
	Lasting more		Sometimes		Seldom or		Lasting more		Sometimes		Seldom or		Lasting more		Sometimes		Seldom or	
	than 2 weeks				never		than 2 weeks				never		than 2 weeks				never	
Characteristics	(n = 15)		(n = 217)		(n = 790)		(n = 24)		(n = 219)		(n = 779)		(n = 35)		(n = 302)		(n = 685)	
Age (y)	45.8±9.1		42.7±9.7		44.2±10.2		47.0±8.5		44.6±9.5		43.6±10.2		48.1±9.7		46.3±9.5		42.6±10.1	
Body mass index (kg/m^2^)	23.9±3.2		24.0±3.4		24.2±3.2		24.3±3.8		24.2±3.3		24.1±3.3		23.9±3.5		24.2±3.2		24.1±3.3	
Sleep duration <6 h	11	(73.3)	115	(53.0)	341	(43.2)	17	(70.8)	118	(53.9)	332	(42.6)	24	(68.6)	141	(46.7)	302	(44.1)
Smoking status																		
Current	3	(20.0)	97	(44.7)	253	(32.0)	3	(12.5)	76	(34.7)	274	(35.2)	5	(14.3)	98	(32.5)	250	(36.5)
Past	8	(53.3)	54	(24.9)	283	(35.8)	16	(66.7)	72	(32.9)	257	(33.0)	20	(57.1)	116	(38.4)	209	(30.5)
Never	4	(26.7)	66	(30.4)	254	(32.2)	5	(20.8)	71	(32.4)	248	(31.8)	10	(28.6)	88	(29.1)	226	(33.0)
Drinking habit																		
Every day	3	(20.0)	77	(35.5)	294	(37.2)	8	(33.3)	95	(43.4)	271	(34.8)	12	(34.3)	139	(46.0)	223	(32.6)
Occasional	7	(46.7)	102	(47.0)	366	(46.3)	12	(50.0)	96	(43.8)	367	(47.1)	15	(42.9)	127	(42.1)	333	(48.6)
Never	5	(33.3)	38	(17.5)	130	(16.5)	4	(16.7)	28	(12.8)	141	(18.1)	8	(22.9)	36	(11.9)	129	(18.8)
Regular physical activity^a^	2	(13.3)	72	(33.2)	337	(42.7)	5	(20.8)	83	(37.9)	323	(41.5)	11	(31.4)	115	(38.1)	285	(41.6)
Family history of diabetes	2	(13.3)	30	(13.8)	101	(12.8)	2	(8.3)	36	(16.4)	95	(12.2)	3	(8.6)	49	(16.2)	81	(11.8)

Data are mean ± SD or n (%). sPercentages may not add up to 100 because of rounding.

a Regular physical activity was defined as continuing at least 30 minutes, 2 times/week, for more than a year.

**Figure 1:**
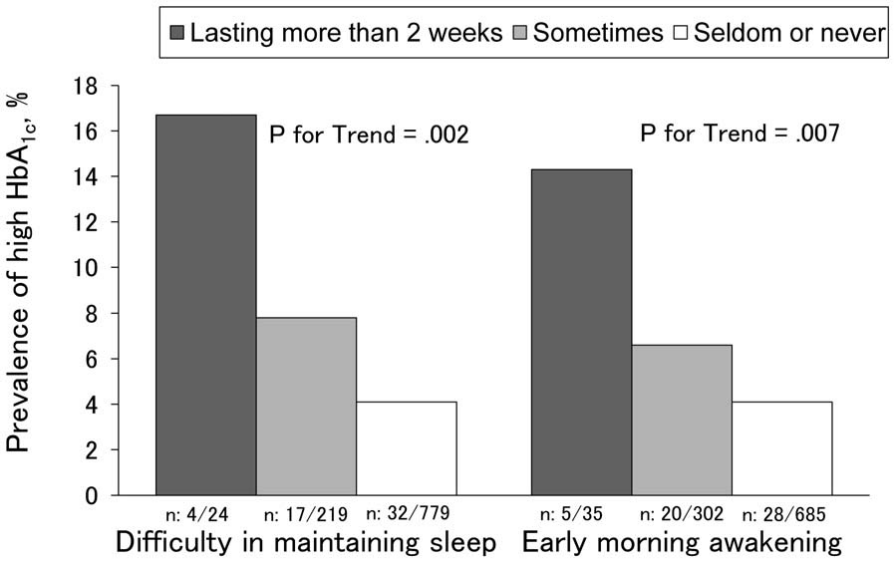
The prevalence of high HbA_1c_ across 3 categories of difficulty in maintaining sleep and early morning awakening. High HbA_1c_ is defined as a blood level of hemoglobin A_1c_ ≥6.0%. P values were obtained from the Mantel-Haenszel chi-square test for linear trend.

The overall prevalence of high HbA_1c_ was 5.2%. There was a significant and graded positive association of difficulty in maintaining sleep (P for trend  = .002) and early morning awakening (P for trend  = .007) with the prevalence of high HbA_1c_ (Figure 1). However, such a linear trend was not observed regarding difficulty in initiating sleep (P for trend  = .953), and none of the participants in the “lasting more than 2 weeks” category of difficulty in initiating sleep had high HbA_1c_.

Table 2 shows ORs of high HbA_1c_ as computed using logistic regression models. In model 1, no significant association was observed between difficulty in initiating sleep and high HbA_1c_. In model 2, participants who experienced difficulty in maintaining sleep lasting more than 2 weeks (adjusted OR 6.79 [95% CI 1.86–24.85]) or sometimes (2.33 [1.19–4.55]) were significantly more likely to have high HbA_1c_, compared with those who experienced this seldom or never. In model 3, participants who experienced early morning awakening lasting more than 2 weeks (3.96 [1.24–12.59]) were significantly more likely to have high HbA_1c_, compared with those who experienced this seldom or never.

**Table 2:** Odds ratios (ORs) for high HbA_1c_ a according to each of 3 types of insomnia symptoms (n = 1,022).

	Model 1	Model 2	Model 3
Variables	OR (95% CI)	OR (95% CI)	OR (95% CI)
Difficulty in initiating sleep			
Lasting more than 2 weeks	N/A	—	—
Sometimes	1.54 (0.75–3.14)	—	—
Seldom or never	1.00	—	—
Difficulty in maintaining sleep			
Lasting more than 2 weeks	—	6.79 (1.86–24.85)*	—
Sometimes	—	2.33 (1.19–4.55)*	—
Seldom or never	—	1.00	—
Early morning awakening			
Lasting more than 2 weeks	—	—	3.96 (1.24–12.59)*
Sometimes	—	—	1.27 (0.67–2.41)
Seldom or never	—	—	1.00
Age (y)	1.11 (1.07–1.15)*	1.11 (1.07–1.15)*	1.10 (1.06–1.14)*
Body mass index (kg/m^2^)	1.27 (1.16–1.38)*	1.27 (1.17–1.39)*	1.27 (1.17–1.39)*
Sleep duration <6 h	0.69 (0.36–1.30)	0.54 (0.28–1.05)	0.60 (0.31–1.15)
Smoking status			
Current	2.46 (0.99–6.12)	2.71 (1.08–6.80)*	2.59 (1.04–6.46)*
Past	2.30 (0.94–5.62)	2.12 (0.86–5.23)	2.08 (0.85–5.11)
Never	1.00	1.00	1.00
Drinking habit			
Every day	1.04 (0.41–2.64)	0.94 (0.37–2.39)	0.99 (0.39–2.53)
Occasional	1.00 (0.40–2.54)	0.88 (0.34–2.24)	0.94 (0.37–2.39)
Never	1.00	1.00	1.00
Regular physical activity^b^	1.35 (0.74–2.46)	1.51 (0.82–2.78)	1.36 (0.75–2.48)
Family history of diabetes	1.84 (0.87–3.89)	1.90 (0.89–4.05)	1.79 (0.84–3.80)

CI, confidence interval. ^*^P<0.05.

A dash (—) indicates that the item was not included in the model.

a High HbA_1c_ was defined as a blood level of hemoglobin A_1c_ ≥6.0%.

b Regular physical activity was defined as continuing for at least 30 minutes, 2 times/week, for more than a year.

Similar associations were observed in multiple regression models as sensitivity analyses (Table S1). After adjusting for all covariates including age, BMI, sleep duration, smoking status, drinking habits, regular physical activity, and family history of diabetes, difficulty in maintaining sleep and early morning awakening lasting more than 2 weeks were found to be significantly associated with continuous HbA_1c_ levels. However, no significant association was observed between difficulty in initiating sleep and continuous HbA_1c_ levels.

## Discussion

We found that difficulty maintaining sleep and early morning awakening were significantly associated with HbA_1c_ level in a dose-response relationship in Japanese men. These associations were independent of age, BMI, sleep duration, smoking status, drinking habit, regular physical activity, and family history of diabetes. The results are consistent with the findings of a previous longitudinal study indicating that difficulty in initiating or maintaining sleep is related to incident diabetes [6] and those of a previous cross-sectional study indicating that poor sleep quality affects glycemic control, as assessed by HbA_1c_ level among patients with type 2 diabetes [9]. The observed dose-response associations suggest that not only insomnia at a clinical level but also milder forms of insomnia have an effect on HbA_1c_ level. The observed dose-response associations also suggest that individuals with greater duration of insomnia symptoms may have higher HbA_1c_ levels. However, no significant association was observed between difficulty in initiating sleep and HbA_1c_ level, possibly due to a lack of statistical power from having a small number of men who reported difficulty in initiating sleep (n = 15). The results revealed that a relatively small number of the participants with a clinical or milder level of any insomnia symptoms had high HbA1c. For example, only four were identified as having high HbA1c among 24 participants with difficulty in maintaining sleep lasting more than 2 weeks, and five among 35 participants with early morning awakening. However, such participants met the clinical DSM-IV-TR criteria for insomnia, and need to be managed appropriately because they had high risks for a variety of mind/body illness including type 2 diabetes and depression. In addition, considering that insomnia is common in today's society, the combined effect of clinical and mild insomnia on type 2 diabetes may be considerable.

Unlike the previous cross-sectional study by Nakajima et al. [10], we observed a significant association between the major symptoms of insomnia and HbA_1c_ level in a Japanese non-patient population. The discrepancy between the 2 studies may be partly due to a difference in the definition of insomnia symptoms. In the previous study [10], the presence of insomnia was defined as symptoms occurring “3 times or more per week,” which was not based on clinical diagnostic criteria such as the DSM-IV-TR.

The mechanisms underlying the association between insomnia symptoms and HbA_1c_ level are unclear. However, several factors associated with insomnia symptoms, including obesity-promoting health behaviors (e.g., reduced energy expenditure and increased food intake) [15] and activation of the sympathetic nervous system and the hypothalamic-pituitary-adrenal axis [16], [17], can cause insulin resistance and increase the risk for diabetes. Additionally, our results showing that men with insomnia symptoms at a clinical level were more likely to be past smokers and that current smoking was significantly associated with high HbA_1c_ provided insight into the mechanism; smoking may confound the association. Smoking is known to be associated with both depression (which is frequently accompanied by insomnia) [18] and type 2 diabetes [19]. Further research is required to explore the underlying mechanisms.

The strengths of our study include a homogeneous study population, a high response rate, accurately defined exposure measurements, and adjustment for important confounders. However, our study has some limitations. First, because of the cross-sectional nature of the study, we could not determine the temporality of the association and exclude the possibility of reverse causality. Indeed, preliminary analysis showed that the participants with previously diagnosed or treated diabetes had a higher prevalence of any insomnia symptoms lasting more than 2 weeks than analyzed participants (10.0% vs. 4.9%). Preliminary analyses also showed more stable associations between insomnia symptoms and high HbA_1c_ levels when participants with previously diagnosed or treated diabetes were included in the analyses (Table S2, Table S3). However, we were able to minimize the possibility of reverse causality by excluding participants with previously diagnosed or treated diabetes. Second, the estimates of OR for high HbA_1c_ need to be interpreted with some caution because of the potential for high variability associated with a small cell size. Third, 3 types of insomnia symptoms assessed in this study are general but limited aspects of insomnia. Impairment in daytime functioning and subtypes based on etiology were not considered. Additionally, insomnia symptoms were self-reported. However, self-reports of insomnia symptoms, as used in this study, are the measures most widely used in epidemiological surveys [6], and the results were confirmed by two specialists in our study. Fourth, although the quantitative aspect of sleep (i.e., short and long sleep durations) is a risk factor for diabetes [6], we could not fully consider the effect of sleep duration on high HbA_1c_ because of the inadequate assessment of sleep duration (i.e., few response options). Fifth, although we considered a range of confounders, unmeasured confounders that may be linked to both insomnia symptoms and diabetes risk could have contributed to the association. Examples of such possible confounders are obstructive sleep apnea syndrome (OSAS) and restless legs syndrome (RLS). OSAS is known to be associated with insomnia [20] and insulin resistance [21]. In Japan, OSAS prevalence in adult males was estimated to be 3.3% [22]. This significant number of OSAS cases might contribute to the association between insomnia symptoms and HbA_1c_ level. RLS is also known to be associated with difficulty in initiating and maintaining sleep and diabetes [23]. However, because RLS prevalence in Asians (0.7%) has been reported to be lower than that in Caucasians (5%–10%) [24], [25], the confounding effect of RLS might be small. Finally, the study population was restricted to men, which limited the generalizability of the findings. A Japanese national survey has shown that men are less likely to report difficulty in initiating and maintaining sleep, but are more likely to report early morning awakening compared to women [1]. Thus, insomnia symptoms might associate differently with HbA_1c_ level depending on the gender. Additionally, the sample of white-collar workers also limits the generalizability of the findings. However, the distribution of each insomnia symptom in our sample was almost identical to that in the general Japanese population [12].

In summary, the present study suggests that insomnia symptoms, particularly difficulty in maintaining sleep and early morning awakening, are closely associated in a dose-response relationship with high HbA_1c_ in Japanese men. Further studies are required to elucidate the association between difficulty in initiating sleep and HbA_1c_ level. Health care practitioners may therefore need to pay more attention to individuals with insomnia symptoms to reduce the risk for diabetes.

## Supporting Information

Table S1 Associations between each of 3 types of insomnia symptoms and HbA_1c_ as a continuous variable.
(DOC)

Table S2 Prevalence of 3 types of insomnia symptoms among analyzed participants and excluded diabetes patients.
(DOC)

Table S3 Associations between each of 3 types of insomnia symptoms and high HbA_1c_ when including diabetes patients in the analysis.
(DOC)
