# Supplementary material for: Association between Insomnia Symptoms and Hemoglobin A1c Level in Japanese Men
Source: PLoS One. 2011 Jul 1;6(7):e21420. doi: 10.1371/journal.pone.0021420 (PMC3128595; doi:10.1371/journal.pone.0021420)
Supplement: Table S1 — Associations between each of 3 types of insomnia symptoms and HbA1c as a continuous variable. (DOC) [file pone.0021420.s001.doc]

| Table S1 Associations between each of 3 types of insomnia symptoms and hemoglobin A_1c_^a^: multiple regression analysis (n = 1,022) | | | | |
| --- | --- | --- | --- | --- |
|  |  | Model 1 | Model 2 | Model 3 |
| Variables | | β (95% CI) | β (95% CI) | β (95% CI) |
| Difficulty in initiating sleep | |  |  |  |
|  | Lasting more than 2 weeks | -0.057 (-0.270–0.156) | — | — |
|  | Sometimes | 0.037 (-0.026–0.100) | — | — |
|  | Seldom or never | Reference | — | — |
| Difficulty in maintaining sleep | |  |  |  |
|  | Lasting more than 2 weeks | — | 0.172 (0.002–0.342)^*^ | — |
|  | Sometimes | — | 0.017 (-0.045–0.080) | — |
|  | Seldom or never | — | Reference | — |
| Early morning awakening | |  |  |  |
|  | Lasting more than 2 weeks | — | — | 0.155 (0.013–0.298)^*^ |
|  | Sometimes | — | — | -0.011 (-0.068–0.046) |
|  | Seldom or never | — | — | Reference |
| Age (y) | | 0.013 (0.010–0.016)^*^ | 0.013 (0.010–0.016)^*^ | 0.013 (0.010–0.016)^*^ |
| Body mass index (kg/m^2^) | | 0.026 (0.018–0.034)^*^ | 0.026 (0.018–0.034)^*^ | 0.026 (0.018–0.034)^*^ |
| Sleep duration <6 h | | -0.010 (-0.062–0.041) | -0.014 (-0.066–0.037) | -0.014 (-0.065–0.038) |
| Smoking status | |  |  |  |
|  | Current | 0.038 (-0.026–0.102) | 0.042 (-0.021–0.106) | 0.043 (-0.020–0.107) |
|  | Past | 0.003 (-0.063–0.068) | -0.005 (-0.071–0.061) | -0.003 (-0.069–0.063) |
|  | Never | Reference | Reference | Reference |
| Drinking habit | |  |  |  |
|  | Every day | -0.052 (-0.128–0.025) | -0.051 (-0.128–0.025) | -0.046 (-0.122–0.031) |
|  | Occasional | 0.006 (-0.066–0.078) | 0.006 (-0.067–0.078) | 0.010 (-0.063–0.082) |
|  | Never | Reference | Reference | Reference |
| Regular physical activity^b^ | | -0.012 (-0.065–0.041) | -0.008 (-0.061–0.044) | -0.011 (-0.063–0.042) |
| Family history of diabetes | | 0.083 (0.007–0.159)^*^ | 0.084 (0.008–0.160)^*^ | 0.086 (0.010–0.162)^*^ |
| β, β coefficient; CI, confidence interval. ^*^P < 0.05. | | |  |  |
| A dash (—) indicates that the item was not included in the model. | | | |  |
| ^a^ Hemoglobin A_1c_ data were entered as a continuous variable. | | | |  |
| ^b^ Regular physical activity was defined as continuing for at least 30 minutes, 2 times/week, for more than a year. | | | | |
