# Supplementary material for: Association between Insomnia Symptoms and Hemoglobin A1c Level in Japanese Men
Source: PLoS One. 2011 Jul 1;6(7):e21420. doi: 10.1371/journal.pone.0021420 (PMC3128595; doi:10.1371/journal.pone.0021420)
Supplement: Table S2 — Prevalence of 3 types of insomnia symptoms among analyzed participants and excluded diabetes patients. (DOC) [file pone.0021420.s002.doc]

Table S2 Prevalence of 3 types of insomnia symptoms among analyzed participants and excluded diabetes patients (n = 1,072)

|  | | Analyzed participants (n = 1,022) | |  | Excluded diabetes patients (n = 54) | |
| --- | --- | --- | --- | --- | --- | --- |
| Insomnia symptoms | | N | % |  | N | % |
| Difficulty in initiating sleep | |  |  |  |  |  |
|  | Lasting more than 2 weeks | 15 | 1.5 |  | 1 | 1.9 |
|  | Sometimes | 217 | 21.2 |  | 11 | 20.4 |
|  | Seldom or never | 790 | 77.3 |  | 42 | 77.8 |
| Difficulty in maintaining sleep | |  |  |  |  |  |
|  | Lasting more than 2 weeks | 24 | 2.4 |  | 2 | 3.7 |
|  | Sometimes | 219 | 21.4 |  | 17 | 31.5 |
|  | Seldom or never | 779 | 76.2 |  | 35 | 64.8 |
| Early morning awakening | |  |  |  |  |  |
|  | Lasting more than 2 weeks | 35 | 3.4 |  | 6 | 11.1 |
|  | Sometimes | 302 | 29.6 |  | 26 | 48.2 |
|  | Seldom or never | 685 | 67.0 |  | 22 | 40.7 |
