# Supplementary material for: Association between Insomnia Symptoms and Hemoglobin A1c Level in Japanese Men
Source: PLoS One. 2011 Jul 1;6(7):e21420. doi: 10.1371/journal.pone.0021420 (PMC3128595; doi:10.1371/journal.pone.0021420)
Supplement: Table S3 — Associations between each of 3 types of insomnia symptoms and high HbA1c when including diabetes patients in the analysis. (DOC) [file pone.0021420.s003.doc]

| Table S3 Odds ratios (ORs) for high HbA_1c_^a^ according to each of 3 types of insomnia symptoms among all participants including diabetes patients (n = 1,072) | | | | |
| --- | --- | --- | --- | --- |
|  |  | Model 1 | Model 2 | Model 3 |
| Variables | | OR (95% CI) | OR (95% CI) | OR (95% CI) |
| Difficulty in initiating sleep | |  |  |  |
|  | Lasting more than 2 weeks | 0.51 (0.06–4.64) | — | — |
|  | Sometimes | 1.26 (0.70–2.27) | — | — |
|  | Seldom or never | 1.00 | — | — |
| Difficulty in maintaining sleep | |  |  |  |
|  | Lasting more than 2 weeks | — | 4.04 (1.24–13.18)^*^ | — |
|  | Sometimes | — | 2.18 (1.30–3.68)^*^ | — |
|  | Seldom or never | — | 1.00 | — |
| Early morning awakening | |  |  |  |
|  | Lasting more than 2 weeks | — | — | 3.90 (1.54–9.85)^*^ |
|  | Sometimes | — | — | 1.46 (0.89–2.39) |
|  | Seldom or never | — | — | 1.00 |
| Age (y) | | 1.14 (1.10–1.17)^*^ | 1.14 (1.10–1.17)^*^ | 1.13 (1.10–1.17)^*^ |
| Body mass index (kg/m^2^) | | 1.26 (1.18–1.35)^*^ | 1.26 (1.18–1.35)^*^ | 1.26 (1.18–1.36)^*^ |
| Sleep duration <6 h | | 0.81 (0.50–1.32) | 0.69 (0.42–1.14) | 0.71 (0.43–1.17) |
| Smoking status | |  |  |  |
|  | Current | 1.85 (0.97–3.53) | 1.93 (1.01–3.71)^*^ | 1.91 (1.00–3.66)^*^ |
|  | Past | 1.57 (0.83–2.95) | 1.45( 0.76–2.73) | 1.42 (0.75–2.67) |
|  | Never | 1.00 | 1.00 | 1.00 |
| Drinking habit | |  |  |  |
|  | Every day | 1.08 (0.53–2.20) | 0.94 (0.46–1.92) | 0.97 (0.48–2.00) |
|  | Occasional | 1.03 (0.50–2.11) | 0.89 (0.43–1.84) | 0.94 (0.46–1.92) |
|  | Never | 1.00 | 1.00 | 1.00 |
| Regular physical activity^b^ | | 1.10 (0.69–1.77) | 1.20 (0.75–1.93) | 1.13 (0.71–1.82) |
| Family history of diabetes | | 3.65 (2.17–6.13)^*^ | 3.67 (2.17–6.19)^*^ | 3.44 (2.04–5.80)^*^ |
| CI, confidence interval. ^*^P < 0.05. A dash (—) indicates that the item was not included in the model. | | | | |
| ^a^ High HbA_1c_ was defined as a blood level of hemoglobin A_1c_ ≥6.0%. | | | |  |
| ^b^ Regular physical activity was defined as continuing for at least 30 minutes, 2 times/week, for more than a year. | | | | |
